# The capsule polysaccharide structure and biogenesis for non-O1 Vibrio cholerae NRT36S: genes are embedded in the LPS region

**DOI:** 10.1186/1471-2180-7-20

**Published:** 2007-03-15

**Authors:** Yuansha Chen, Peter Bystricky, Jacob Adeyeye, Pinaki Panigrahi, Afsar Ali, Judith A Johnson, CA Bush, JG Morris, OC Stine

**Affiliations:** 1 Department of Epidemiology and Preventive Medicine, University of Maryland, Baltimore, Baltimore, MD 21201, USA; 2 Department of Chemistry and Biochemistry, University of Maryland, Baltimore County Baltimore, MD 21250, USA; 3 Department of Pediatrics, University of Maryland, Baltimore, Baltimore, MD 21201, USA; 4 Department of Pathology, University of Maryland, Baltimore, Baltimore, MD 21201, USA; 5 Clinical Microbiology, Veterans Affairs Maryland Health Care System, Baltimore, MD 21201, USA; 6 Slovak Medical University; Bratislava, SK-83303, Slovakia; 7 Department of Natural Sciences, Coppin State College, Baltimore, MD 21216, USA

## Abstract

**Background:**

In *V. cholerae*, the biogenesis of capsule polysaccharide is poorly understood. The elucidation of capsule structure and biogenesis is critical to understanding the evolution of surface polysaccharide and the internal relationship between the capsule and LPS in this species. *V. cholerae *serogroup O31 NRT36S, a human pathogen that produces a heat-stable enterotoxin (NAG-ST), is encapsulated. Here, we report the covalent structure and studies of the biogenesis of the capsule in *V. cholerae *NRT36S.

**Results:**

The structure of the capsular (CPS) polysaccharide was determined by high resolution NMR spectroscopy and shown to be a complex structure with four residues in the repeating subunit. The gene cluster of capsule biogenesis was identified by transposon mutagenesis combined with whole genome sequencing data (GenBank accession DQ915177). The capsule gene cluster shared the same genetic locus as that of the O-antigen of lipopolysaccharide (LPS) biogenesis gene cluster. Other than *V. cholerae *O139, this is the first *V. cholerae *CPS for which a structure has been fully elucidated and the genetic locus responsible for biosynthesis identified.

**Conclusion:**

The co-location of CPS and LPS biosynthesis genes was unexpected, and would provide a mechanism for simultaneous emergence of new O and K antigens in a single strain. This, in turn, may be a key element for *V. cholerae *to evolve new strains that can escape immunologic detection by host populations.

## Background

*Vibrio cholerae *has three forms of surface polysaccharide, although some strains do not express all three forms: a lipopolysaccharide (LPS) inserted in the outer membrane, a capsule composed of high molecular weight polysaccharide that forms a dense thick coat outside of the bacterial cells, and a loose slime-like exopolysaccharide. Unlike *V. cholerae *of serogroup O1, which causes cholera, most non-O1 isolates have capsular polysaccharide (CPS) in addition to LPS. The LPS of *V. cholerae *is a protective antigen for cholera [1,2], with over 200 serogroups identified based on the O-antigen of the LPS. The O-antigen biogenesis loci of 4 serogroups (O1, O139. O22, O37) have been sequenced and characterized, and have been found to reside between two genes, *gmhD *and *rjg*, in the genome [3-7]. More than 85% of non-O1 *V. cholerae *isolates have a capsule that is critical for virulence in extraintestinal infections [8]. However, in contrast to *E. coli*, in which extensive work has been done on capsule structure and genetics (with associated classification into groups by Whitfield and Roberts [9]), structures and the genetics of CPS in *V. cholerae *are poorly understood.

The one strain for which data on capsule structure and genetics are available is the newly emerged epidemic strain *V. cholerae *O139. This strain has a capsule that appears to have arisen from the replacement of the O1 antigen biosynthetic region with a new gene cluster in the genetic background of an O1 strain [5,6], resulting in emergence of a strain to which the human population did not yet have immunity. The capsule in O139 is unusual in that it shares the same repeating subunit as the O-antigen [10-12]. Therefore, the polysaccharide in O139 appears as both capsule and LPS and resembles the K_LPS _in the group 4 *E. coli *capsule [9].

There are limited studies on the genetics of polysaccharide biogenesis for the genus *Vibrio*. In *V. vulnificus *the CPS is a primary virulence factor and hence has been the target of more intensive study [13]. An operon including genes *wza*, *wzb *and *wzc *was identified as part of the CPS genes for *V. vulnificus *strain M06-24 [14,15], consistent with the presence of a group 1 capsule. The genetic loci for CPS were also identified in another strain of *V. vulnificus *1003 [16]. A wzx/wzy system was present for polymerization and exporting the CPS. However, the genetic region responsible for LPS biosynthesis has not been identified in *V. vulnificus*.

The elucidation of capsule structure and biogenesis is critical to understanding the evolution of surface polysaccharide and the internal relationship between the capsule and LPS in this species. It also has clear implications for understanding the behavior of this species within human populations, as the ability to change these surface antigens to avoid host immunologic detection is a key feature underlying the ability of *V. cholerare *to survive. Here, we report the covalent structure and studies of the biogenesis of the capsule in *V. cholerae *NRT36S.

## Results and discussion

### Structure of the CPS

#### Gas chromatography

Carbohydrate analysis was done by gas chromatography of the trimethyl silyl methyl glycosides and the absolute configurations were determined by gas chromatography of the + and - 2-butyl glycosides as the trimethyl silyl derivatives. Both experiments were performed at the Complex Carbohydrate Research Center (CCRC) at the University of Georgia. The results indicated L-rhamnose (Rha), D-glucosamine (GlcNAc), D-glucuronic acid (GlcA) and D-galactose (Gal). The results of methylation analysis, also performed at CCRC, are given in Table 1. These results indicate that the major components of the hydrolyzed sample of methylated CPS sample represent 3-linked L-rhamnose, 4-linked D-glucosamine, 4- and 6-linked D-glucosamine and galactose in various linkages including 3-linked, 4-linked and 3,4 -linked. The D-glucuronic acid residue cannot be detected by this analysis because the uronic acids are converted to a sodium salt in the standard protocol [17].

#### Nuclear magnetic resonance (NMR) results

NMR spectra of the native polysaccharide are complex, showing a number of peaks in the anomeric region that are not in simple stoichiometric ratios. Likewise, the acetyl methyl region (2.0 – 2.3 ppm) shows approximately 10 peaks not in simple ratios suggesting that the polysaccharide may be heterogeneously substituted with O-acetyl functions. Therefore the sample was treated with aqueous ammonium hydroxide, which is expected to cleave O-acetyl groups by mild base catalyzed hydrolysis. The NMR spectra of the resulting sample (de-O-acetyl polysaccharide) showed only two peaks in the acetyl methyl region (2.06 and 2.09 ppm) and a greatly simplified pattern in the anomeric region with four distinct signals in the C-H HSQC (heteronuclear single quantum correlation) spectrum. We show below that the peaks at 2.06 and 2.09 arise from N-acetyl groups.

**Figure 1:**
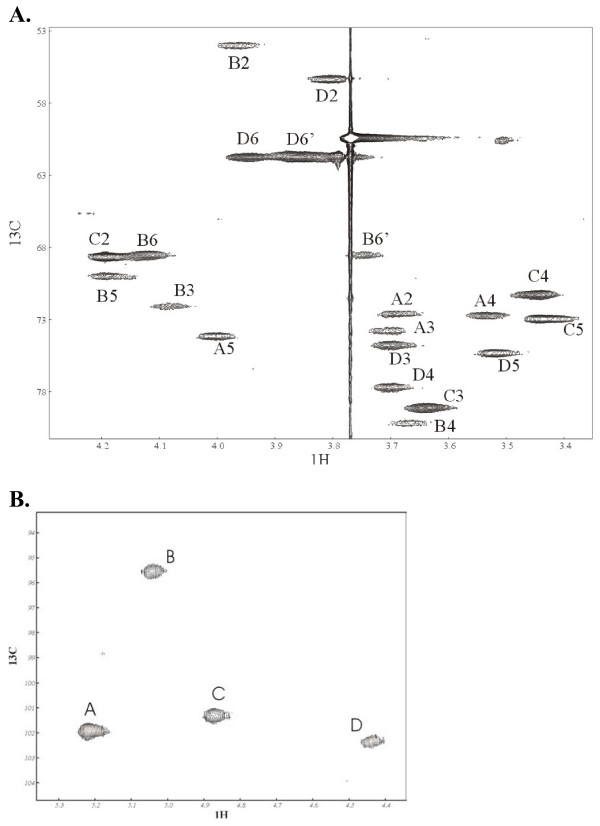
Panel A shows the HSQC spectrum of the de-O-acetylated capsular polysaccharide from NRT36S. The strong signal at 3.78, 60 ppm is a low molecular weight impurity. Panel B shows the signal from the anomeric region. The methyl group region is not shown.

**Figure 2:**
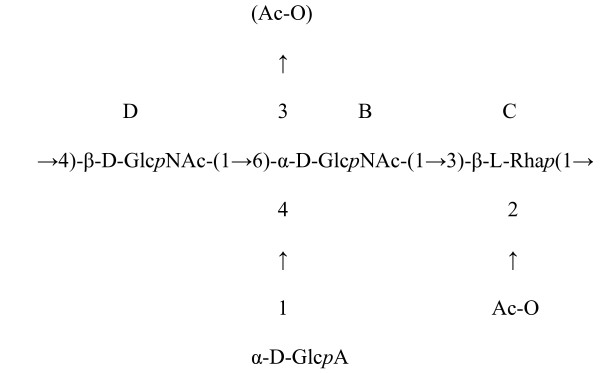
Proposed structure of NRT36S CPS repeating unit. Parentheses indicate partial O-acetylation of residue B at the 3-position.

The HSQC spectrum (Figure 1A&1B) indicates four sugar residues in the repeating subunit of the polysaccharide and the four signals were arbitrarily assigned identifying letters, A, B, C and D for the purpose of individual sugar identification using homonuclear ^1^H spin correlation. Experiments used to identify the sugar ring spin systems included COSY (correlation spectroscopy), TOCSY (total correlation spectroscopy), HMBC (heteronuclear multiple bond coherence) and NOESY (nuclear Overhauser spectroscopy). Residue C is identified as rhamnose by the characteristic methyl resonance of the 6-deoxy sugar at 1.33 ppm in combination with the equatorial configuration of H2 indicated by its small homonuclear coupling constants. The anomeric configuration is identified as β – by large NOESY peaks between H1, H3 and H5 as well as by ^1^J_CH _= 162 Hz. Residues B and D are identified as amino sugars by the characteristic chemical shift of C2 and as glucosamine by homonuclear coupling values. Residue B has the α-anomeric configuration as indicated by small J_H1-H2 _and by ^1^J_CH _= 172 Hz while residue D has the β – configuration as indicated by large J_H1-H2 _and by ^1^J_CH _= 158 Hz. The fourth residue, A, is identified as α-glucuronic acid. ^1^J_CH _for the anomeric signal is 168 Hz. and J_H1-H2 _is small, but the coupling constants of H3, H4 and H5 are all large as expected for the gluco configuration. No crosspeaks can be detected for H6 but an HMBC spectrum selective for the carbonyl region shows crosspeaks between both H4 and H5 and a carbonyl resonance at 176.7 ppm consistent with α-glucuronic acid. The central part and the anomeric region of the HSQC spectrum of the de-O-acetyl polysaccharide are shown in Figure 1A&1B and the complete ^1^H and ^13^C resonance assignments are given in Table 2. The glycosidic linkages between the four residues were determined from HMBC and NOESY data as indicated in Table 3. While no HMBC peaks could be observed for the D-B linkage, the nuclear Overhauser data clearly indicate a β-1–6 linkage and the downfield chemical shift of B-C6 at 68.9 ppm confirms this linkage assignment. The proposed structure of the tetrasaccharide repeating unit is given in Figure 2.

Having determined the structure of the sugar backbone, we turned to the acetylated forms of the polysaccharide. While interpretation of their complex spectra was made difficult by heterogeneity, it was possible given the basic sugar structure. Base hydrolysis milder than that required to produce the de-O-acetyl polysaccharide yielded a sample with NMR spectra having peaks in stoichiometric ratios (mono-O-acetyl polysaccharide). The single O-acetyl group is assigned by carbonyl-selective HMBC to the 2-position of rhamnose (C), a position resistant to base hydrolysis due to the absence of a neighboring hydroxyl function. A complete set of homonuclear and heteronuclear NMR spectra of a sample of this form of the polysaccharide in H_2_O solution allowed assignment of signals of the amide protons of residues B and D confirming that they are N-acetyl amino sugars. The complete assignment of the NMR spectrum of the mono-O-acetyl polysaccharide is given in Table 4.

The acetate methyl region of the NMR spectrum of the native, untreated, polysaccharide shows a number of peaks including those assignable to the amides of residue B and D and of the 2-O-acetyl group of residue C along with smaller peaks indicating partial O-acetylation at other positions. Using HMBC spectra, it was possible to correlate methyl proton signals, through carbonyl carbon resonances, to sugar ring protons indicating positions of acetyl substitution. In addition to the 2-O-acetyl of residue C, it was possible to identify an O-acetyl group on the 3-position of residue B to the extent of about 50% as indicated in Table 4. Anomeric resonances of residue A were split into three peaks in the native polysaccharide suggesting partial O-acetylation of that residue but the exact positions could not be definitively assigned.

Although the chromatographic data indicate that the sample contains galactose, it is not part of the capsular polysaccharide. The NMR data of Figure 1 are consistent with four residues per repeating subunit and no sign of any galactose. Although the NMR spectra show minor peaks at approximately 10% level, none are characteristic of carbohydrates and they show no connection to the peaks assigned for the polysaccharide structure by NOESY or HMBC spectra.

The structure proposed here for the NRT36S capsule repeating subunit is very similar to that reported for the *V. cholerae *O6 lipopolysaccharide [18]. The polysaccharide backbones are identical differing only in the degrees of O-acetylation. Bergstrom et al reported stoichiometric O-acetylation at both C2 of rhamnose and at C3 of α-GlcNAc while our structure for native capsular polysaccharide of *V. cholerae *NRT36S is only partially substituted at C3 of α-GlcNAc along with lower degrees of acetylation at other positions. In spite of these differences, the NMR data reported by Bergstrom et al [18] are quite close to those reported for our native structure in Table 4.

### Transposon mutagenesis and mutant selection

The conjugations between wild type *Vibrio cholerae *NRT36S and donor strain *E. coli *S17λpir/putKm-2 generated 20,615 mutants of NRT36S, each carrying a single copy of the transposon Km-2 in its genome. Among these mutants, 411 colonies displayed a translucent phenotype on LB agar. This phenotype suggests that genes involved in capsule biogenesis have been disrupted by the transposon [19].

### DNA analysis of mutants

Genomic DNA was isolated from the translucent mutants and analyzed by inverted polymerase chain reaction (PCR) and sequencing, identifying 13 unique insertion sites in 11 genes. Since NRT36S can also undergo spontaneous phase variation between transluscent and opaque colony morphologies, isolates with the 13 insertions were tested for complement resistance [19]. Nine insertions in eight genes were sensitive to serum killing and showed no reversion to the opaque morphology (Table 5). Isolates with insertions in the other 3 genes reverted to opaque colonies and were resistant to serum killing and therefore were excluded from further analysis. Only four of the stable mutant genes related to sugar modification and processing and were considered as putative structural genes for the biogenesis of the NRT36S capsule. The function of the other genes was unclear. One of the putative structural genes had a homolog in the fully sequenced genome of *V. cholerae *N16961, a serogroup O1 pandemic strain. Orf23, a homolog of VC0262, an UDP-glucose 4-epimerase (*galE*) was disrupted in translucent colony TR3. TR3 was restored to opaque phenotype and resistant to serum killing when complemented with gal *E *gene. The other three structural genes identified by transposon mutagenesis did not have homologs in the genome of *V. cholerae *N16961. In translucent colony TR17, a glycosyltransferase gene was disrupted and a rhamnosyltransferase gene was disrupted in both TR43 and TR287. An ABC transporter system integral membrane protein gene *wzm *was disrupted in TR296. The VC0262 homolog and the 3 other genes are typical of genes commonly found in polysaccharide biogenesis.

### Immuno blotting and size exclusion chromatography (SEC)

**Figure 3:**
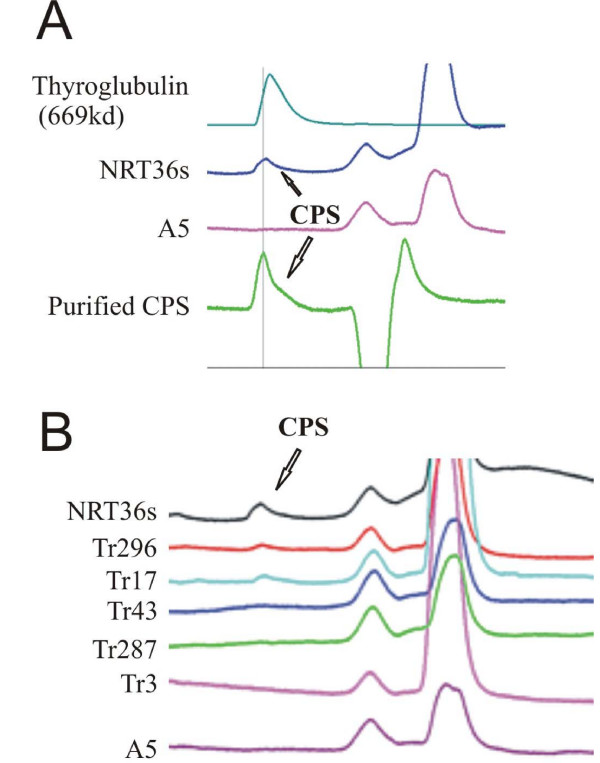
Size exclusion chromatography of the capsule prep. A). Capsule prep from control (NRT36S and A5) compared to purified CPS of NRT36S. The size was estimated by thyroglobulin. The arrow indicates the peak of the capsule at about 13.2 minutes retention time, which corresponds to about 670 k Dalton molecular weight. B). Capsule prep from mutants and control.

**Figure 4:**
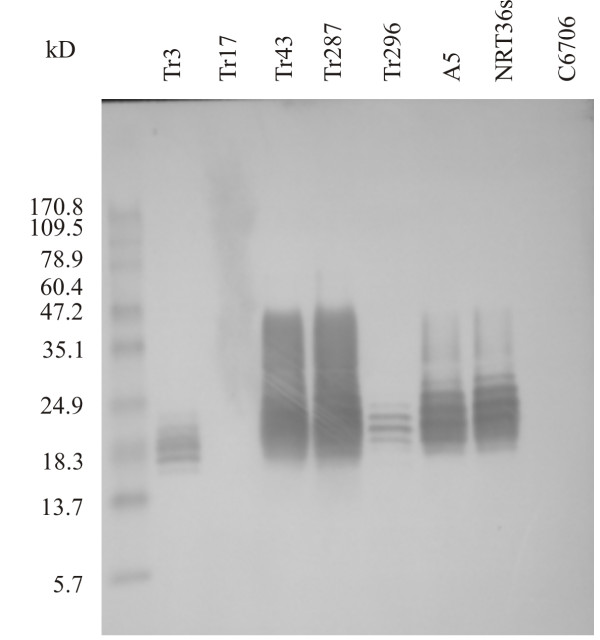
Immuno blot of NRT36S antiserum against external polysaccharide preparations from various strains.

SEC data indicated that the molecular weight of the NRT36S capsule is greater than 670k Dalton (Figure 3A&3B). The antiserum raised against the whole cell of *V. cholerae *NRT36S did not detect anything close to that molecular weight of CPS in the immuno blot (Figure 4), indicating that the antibodies did not react with the capsule. This result was consistent with the previous finding [20]. Nevertheless, the antibodies detected some polysaccharides that formed a ladder pattern in the molecular weight range of 20k to 40k Dalton (Figure 4). We believed these were the LPS. Interestingly, the amount of reactive LPS to the antibody was reduced in mutants TR3, TR17, and TR296. Analysis of the capsule preps by SEC from the mutants showed that three of the mutants, TR3, Tr287 and TR43 had completely lost the high molecular weight peaks corresponding to the capsule while in TR17 and TR296, the amount of capsule was significantly reduced (Figure 3B).

### Electron microscopy (EM)

**Figure 5:**
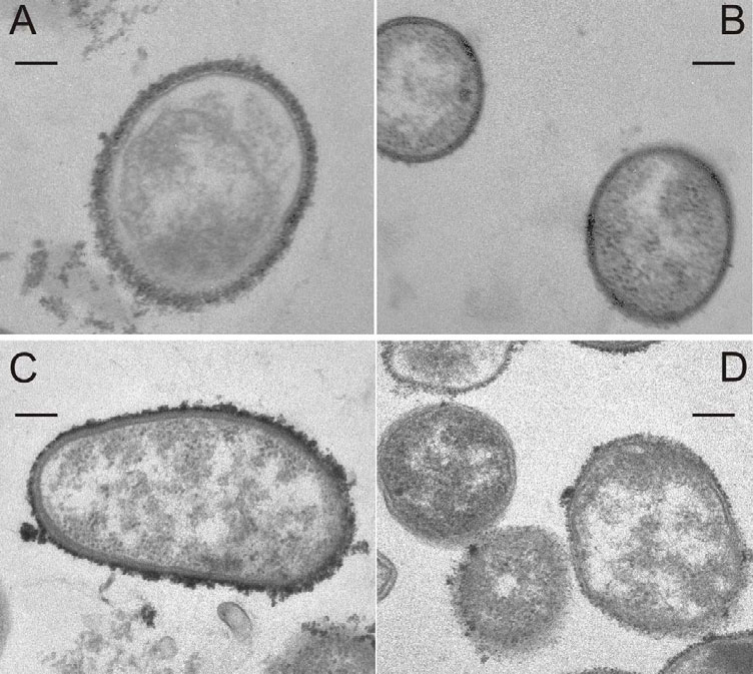
Thin sections of *V. cholerae *NRT36S and its translucent mutants stained with polycationic ferritin. (A) NRT36S; (B) TR3; (C) TR296; (D) TR17. Bar, 200 nm.

We evaluated thin sections of wild type *V. cholerae *NRT36S and several translucent mutants stained with polycationic ferritin by EM. Representative profiles are shown in Figure 5. As seen before ([19], NRT36S displayed a heavy, complete capsule surrounding the cell (Figure 5A). TR3 did not have a complete capsule, but had some patches of capsule materials (Figure 5B). Both of TR17 and TR296 had a much thinner capsule compared to opaque NRT36S (Figure 5C&5D). EM pictures for all three mutants were consistent with the amounts of capsule observed by SEC (Figure 3B).

### Sequencing of the *V. cholerae *NRT36S genome

*V. cholerae *NRT36S genome was sequenced by the company 454 Life Sciences. The sequencing runs have generated 1,082,967 reads and output 104,531,256 bases of sequence. The estimated coverage depth was 26X. The draft genome consisted of 184 contigs with total length of 3.9 million bases. The average GC (guanine-cytosine) content for the draft genome was 47.5%. The draft genome was annotated [21,22]. For the purpose of the discussion in this paper, only those features related to the polysaccharide biogenesis will be discussed.

### Genetics of the polysaccharide biogenesis

#### O-antigen region

**Figure 6:**
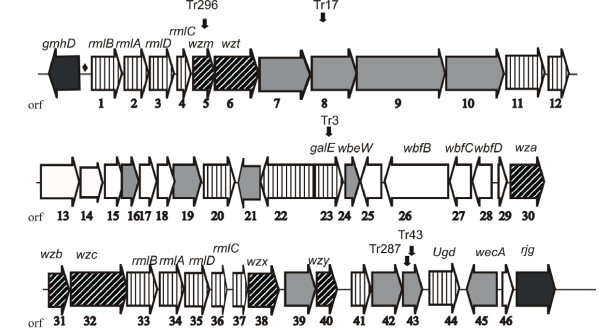
Map of the CPS/O-antigen region of *V. cholerae *NRT 36s. JUMPstart site is indicated by a diamond. Transposon insertion sites are indicated by black arrows. Design patterns of open reading frames indicate different classes of genes: vertical lines, pathway genes; diagonal lines, processing and transportation genes; grey box, glycosyltransferase; white box, functions not clear.

In previous studies, the O-antigen biogenesis genes for *V. cholerae *had been identified to cluster at one locus in the genome, between genes *gmhD *and *rjg *[3,4,6,23]. After aligning the contigs of the draft genome of *V. cholerae *NRT36S to the fully sequenced genome of *V. cholerae *N16961 [24], we found that 3 contigs (contigs 34, 19, 78) of NRT 36S can partially align to the O-antigen region of N16961. Contig 34 contains *gmhD *gene and contig 78 contains *rjg *gene. Contig 19 falls into the middle of contig 34 and 78. The sequence information from analyzing the transposon mutagenesis mutants was able to pick up two more contigs (contig 98 and 43) and connect them to contig 19. Therefore *gmhD *and *rjg *were separated by 5 contigs in the NRT36S genome. The gaps between these contigs were filled and we ratified this region, between *gmhD *and *rjg*, as the putative O-antigen biogenesis region (Figure 6) for *V. cholerae *NRT36S. The sequence between *gmhD *and *rig *was deposited into GenBank (accession DQ915177).

#### CPS region

We located the capsule biogenesis genes identified by transposon mutagenesis in the NRT36S genome (Table 5). To our surprise, the 4 putative capsule structural genes identified by transposon mutagenesis were all located between the genes *gmhD *and *rjg*, the region considered to encode O-antigen biogenesis (Figure 6). Theses four genes were knocked out by at least one of 5 independent transposition events and caused the translucent phenotype associated with the loss of the capsule in each case. Therefore, we believe that the O-antigen biogenesis region in *V. cholerae *NRT36S is also the capsule biogenesis region.

#### Global features

The locus of CPS/O-antigen was 49,916 base pairs in length between genes *gmhD *and *rjg*. There were 46 open reading frames (orf) (Figure 6). The annotation for each orf is listed along with a match from Genbank and its percent amino acid identity/positive, the species and the E-value (Table 6). Twelve genes were glycosyltransferases, 16 genes were recognized as pathway genes for synthesis of the nucleotide sugar precursor for external polysaccharide, and 6 other genes were recognized as polysaccharide processing and translocation genes. The function of the other 12 genes was unknown. A JUMPstart site [25] was located just downstream from the *gmhD *gene. The GC content of this region was 41.2%, lower than the 47.5% GC content of the genome. The disruption by transposon in orf5 (*wzm*), an ABC transporter gene, orf8, a glycosyltransferase gene, orf23, an UDP-glucose-epimerase, (*galE*) and orf43, a rhamnosyltransferase gene had caused the non-encapsulation of NRT36S (Figure 3). The complementation by *galE *gene reverted the translucent mutant TR3 to opaque phenotype and the complemented colonies were resistant to serum killing.

#### Glycosyltransferases

There were 12 glycosyltransferase genes identified. The precise function of most of them remained to be elucidated. Orf45 (*wecA*) was an undecaprenylphosphate N-acetylglucosamine 1-phosphate transferase gene. WecA was putatively the initial transferase to catalyze the transfer of N-acetylglucosamine 1-phosphate to undecaprenylphosphate in the capsule polysaccharide synthesis.

#### Synthesis genes

The structural data for *V. cholerae *NRT36S indicate that the capsule contains, one residue each of rhamnose and glucuronic acid and two N-acetyl-glucosamine residues, genes for whose synthesis are present in the CPS region. There are two sets of genes that are almost identical (orf1-4, and orf33-36) for L-rhamnose synthesis; they were *rmlB*, *rmlA*, *rmlC*, and *rmlD *in the order. L-rhamnose is commonly present in bacterial polysaccharides and the genes to synthesize it are normally clustered. For example in *V. cholerae *isolates with LPS serotypes O6, O12, O14, O19 and O151, the 4 orfs following *gmhD *are *rmlB*, *rmlA*, *rmlC*, and *rmlD *[26]. Orf43 may be the rhamnosyltransferase to catalyze the addition of rhamnose to the CPS backbone. The disruption orf43 by the transposon both resulted in the loss of the capsule. This observation is consistent with the presence of rhamonse in the repeating polysaccharide backbone of the capsule. Orf23 was gene *galE*; its product UDP-glucose 4-epimerase catalyzed the conversion of UDP-glucose to UDP-galactose. Disruption of *galE *gene in mutant TR3 caused the loss of the capsule. Orf24 (*wbeW*) transfers galactose to the capsule complex. Orf 41, a sugar O-acetyltransferase homologue could be involved in the observed O-acetylation of the capsule, but this modification of bacterial polysaccharides is not well understood and other genes may be involved as well. Orf11, 12, 22, 37, 41 and 44 were also putative pathway genes for the synthesis of nucleotide sugar precursors, but their precise functions were not clear to us.

#### Translocation and processing genes

An ABC-2 type transporter system consisted of *wzm *and *wzt *were present in the CPS/O-antigen region. When *wzm *was disrupted by transposon mutagenesis, the mutant was non-capsulated (Table 5). Orf38 was predicted as a polysaccharide translocase gene *wzx*. Orf40 was predicted to have several transmembrane domains by the Dense Alignment Surface (DAS) program [27] and were assigned as putative wzy. Three genes *wza*, *wzb *and *wzc *were also present in the CPS/O-antigen region. The proteins Wza, Wzb and Wzc in *E. coli *formed a system that was involved in the exportation of *E. coli *group 1 capsular polysaccharides [28].

The disruption of an ABC transporter system integral membrane protein gene *wzm *had significantly reduced the amount of capsule (Figure 3B) in our experiments and resulted in the translucent colonies that were susceptible to serum killing. Examination of the CPS region also revealed the existence of *wzt*, which is another component of the ABC transporter system. Our results suggest that the processing and translocation of the capsule in *V. cholerae *NRT36S involves the ABC transporter system. There was a recent report that an ABC transporter system was involved in the transportation of hetero-polysaccharides in the O-antigen of *E. coli *O52 [29]. Our results may be another case where an ABC transporter system was involved in the transportation of a hetero-polysaccharide.

### Sharing of the same region by CPS and LPS

The O-antigen genes had been identified for several serogroups in *V. cholerae*, including O1, O139, O22 and O37 [3,4,6,7,23]. In these serogroups, the gene cluster for O-antigen biogenesis all resided between the genes *gmhD *and *rjg*. In our study of *V. cholerae *NRT36S genome, there was a gene cluster identified as the LPS core biogenesis region upstream of the *gmhD *gene (data not shown). There was also another gene cluster in the genome that was identified as the rugose-associated exopolysaccharide biosynthesis region (data not shown). The homologs of these genes were recognized as exopolysaccharide genes that related to the rugose phenotype in *V. cholerae *O1 El Tor [30]. Besides these regions, i. e., the LPS core genes and the rugose exopolysaccharide genes, there were no other significant gene clusters for polysaccharide synthesis in the genome of *V. cholerae *NRT36S. All of this evidence supported the conclusion that the CPS region, i.e. the region between *gmhD *and *rjg *genes, is indeed also the O-antigen gene cluster. The immuno blot showed that the LPS had been altered in the non-encapsulated mutants. That not only confirmed the sharing of the CPS and LPS region, but also indicated that some genes may be shared by the biogenesis of the two polysaccharide structures. A potential test of this suggestion would be to determine whether the genes other than those encoding rhamnose synthesis involved in the O6 LPS serotype are related to the genes in NRT36S since the two organisms have LPS and capsule structures that are nearly identical.

**Figure 7:**
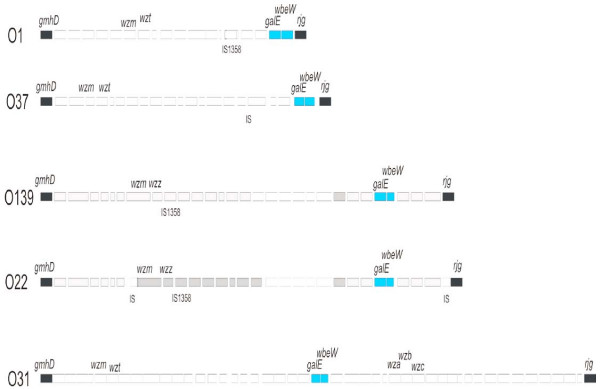
Pictorial representation of the genes in the LPS/CPS regions of O1, O37, O22, O139 and O31 *Vibrio cholerae*. The *galE *and *wbeW *genes found in common between all five sequences are marked in blue. The housekeeping genes *gmhD *and *rjg *that delineate the region are marked in black. The genes in common between O22 aand O139 are marked in grey. The transport genes and the IS elements are labeled. The unlabeled genes represented by white boxes are not found in common across the regions.

The known O-antigen and now CPS regions from *V. cholerae *shared remarkably few genes. The sequences between *gmhD *and *rjg *are known for O1, O22, O37, O139 and now O31 (Figure 7). Only the three genes are found in all five sequences *wzm*, *galE *and *wbeW*. Our mutagensis experiments revealed that both *wzm *and *galE *were essential for capsule production, but enigmatically O1, although it has the genes, it does not have a capsule. Galactose is part of the polysaccharide backbone in O139 for both the LPS and the CPS, but it is not part of the backbone in either O1 LPS or O31 CPS. The structure of O37 is not known. Of note, the O31 region is the first not to contain an IS element.

CPS and O-antigen shared the same genetic locus in *V. cholerae *NRT36S. This differs from the organization of CPS and O-antigen gene clusters in *E. coli*. In *E. coli*, CPS gene clusters and the O-antigen gene clusters are different [9]. The CPS genes from other gram-negative bacteria including *Haemolyticus influenza*, *Salmonella typhi *and *Neisseria meningitidis *have been cloned and characterized [31-36]. The organizations of most of them resemble *E. coli*. No LPS genes have been reported to embed inside the CPS gene cluster for these species except in *N. meningitides*. In *N. meningitides *group B, the mutations of two CPS biosynthesis genes (*synX *or *synC*) and another gene next to the CPS region, *galE *gene, were shown to affect the lipooligosaccharide structure [33,36]. The CPS and O-antigen in group 4 *E. coli *consist of the same molecule. This arrangement is also seen in *V. cholerae *O139 where CPS and O-antigen are encoded by the same genetic locus and have identical repeating subunits. NRT36S is an O31 serogroup. Kondo's group found that the LPS of O31 in *V. cholerae *contains L-glycero-D-mannoheptose, glucose, fructose, galactose, glucosamine and an unknown amino sugar A2 [37]. L-rhamnose was not found in the LPS, while our study found L-rhamnose in the CPS, suggested that CPS and LPS are two distinct structures in *V. cholerae *NRT36S. The antiserum against NRT36S detected LPS but not the capsule, again suggested that the O-antigen and CPS were two different entities. Our finding represents a new type of genetic organization of polysaccharide genes and raises a question of differential regulation of the genes for expression of capsule and O-antigen polysaccharides.

## Conclusion

The genes for bacterial surface polysaccharide biogenesis were typically found in a cluster with an atypical GC content compared to the rest of the genome [38]. It had been suggested that bacteria could convert to a new serogroup by acquiring a new O-antigen biogenesis region. There was abundant evidence that *V. cholerae *O139 arose from an O1 strain by receiving a new O-antigen gene cluster [5,39,40]. The sharing of CPS and O-antigen in *V. cholerae*, as indicated in our findings, makes this region one interchangeable unit. It is possible that *V. cholerae *can acquire a new gene cluster and give rise to a new strain with an O-antigen and K-antigen at the same time, both unrecognizable to the host immune system. This, in turn, may be a key element in its ability to survive, permitting rapid emergence of new strains that can escape immunologic detection by host populations.

## Methods

### Bacterial strains and culture conditions

*V. cholerae *NRT36S is an isolate originally cultured from a Japanese patient with travelers' diarrhea. It is serogroup O31, cholera toxin negative and produces a heat stable enterotoxin NAG-ST [41]. When fed to volunteers this strain caused diarrhea, including, in one patient, a 5.3-liter diarrheal purge [5]. Wild type *V. cholerae *NRT36S produces a capsule [19]. This strain is resistant to polymyxin B but sensitive to kanamycin.

Transposon mini-Km2 was carried in the plasmid putKm-2 and maintained in the host strain *E. coli *S17λpir. Mini-Km2 was found to randomly transpose into the genome of the recipient strain with a single transposition. Mini-Km2 encodes a gene for kanamycin resistance. [42,43]

Cultures were maintained in L broth with 15% glycerol at -70°C. Bacteria were cultured in broth or agar at 37°C unless otherwise stated. Appropriate antibiotics were added in concentrations: 50 mg/ml kanamycin, 50 unit/μl polymyxin B.

### Isolation and purification of CPS

Frozen stock of NRT36S was streaked for isolation on L agar in 150-mm Petri dishes and incubated overnight. A single bacterial colony from the plate was inoculated into 10 ml of L-broth for 18 h of growth. One ml of the culture was then inoculated into 1-liter L-broth and incubated overnight. Bacterial cells from eight 1-liter batches of culture were pelleted at 10,000 g and resuspended in 120 ml of 0.5X phosphate-buffered saline (PBS) pH 7.5 and shaken at 200 rpm on a rotary shaker for 2 h at room temperature. The bacterial suspensions were centrifuged to remove cell debris at 16,000 g for 20 minutes at 4°C and the supernatant dialyzed with multiple changes of distilled water and concentrated two fold by ultra filtration (100,000-nominal-molecular-weight stirred cell; Amicon, Beverly, Mass). The retentates were then ultra centrifuged at 154,000 g for 2 h at 20°C and the supernatants were removed and digested with RNase A (100 ug/ml) and DNase 1 (50 ug/ml plus 1 mM MgCl_2_) for 2 h followed by a 3 h digestion with proteinase K(250 ug/ml) and phenol-chloroform extraction. The aqueous layer was dialyzed as described above, and the resultant sample was lyophilized. Purity of the CPS was assessed by bicinchoninic acid protein assay (MicroBCA, Pierce Chemical Co., Rockford Ill.), and Limulus amoebocyte lysate assay (Sigma Chemical Co., St. Louis, Mo.) and by NMR.

### Deacylation and preparation of samples for NMR

A sample of 15 mg of CPS in 0.6 ml D_2_O was subjected to deacylation by 0.3 M ammonium hydroxide for 6 days at 25 C. The 1D ^1^H spectrum was monitored daily and the sample was lyophilized, exchanged with 99.9% D_2_O and made up with 0.6 ml pure D_2_O (de-O-acetyl polysaccharide). A second sample was prepared with milder base treatment in 0.03 M ammonium hydroxide for 5 days at 4°C (mono-O-acetyl polysaccharide). For detection of amide protons, a sample was subjected to deacylation (without D_2_O exchange) and made up with 0.6 ml 90:10 (v/v) H_2_O:D_2_O.

### NMR spectroscopy

NMR experiments were performed on Bruker 500 MHz DRX AVANCE spectrometer equipped with cryoprobe, with a Bruker 800 MHz DMX AVANCE spectrometer. All Bruker data were processed with NMRPipe and NMRDraw under Linux. All spectra were taken at 50°C due to dynamic properties of the polysaccharide. The following experiments were recorded using standard sequences: 1D: ^1^H spectrum, ^13^C spectrum; 2D: ge-DQF-COSY (E/A), ge-TOCSY (DIPSI-2 mixing times 30 and 70 ms, E/A), ge-NOESY (mixing time 50 ms), ge-HSQC (decoupled, E/A), ge-HMBC (long-range evolution time 30 ms, magnitude mode), HSQMBC (long-range evolution time 15 ms), ge-HMQC-COSY (E/A), ge-HSQC-TOCSY (decoupled, DIPSI-2 mixing time 30 ms, E/A). Also the direct ^31^P and 1D ^31^P spin-echo difference spectra were measured. For detection of amide protons, the following experiments were recorded on a sample of mono-O-acetyl polysaccharide in 90% H_2_O/10% D_2_O solution: 1D: ^1^H spectrum; 2D: ge-TOCSY (DIPSI-2 mixing time 50 ms, E/A, WATERGATE 3–9–19 combined with presaturation). All chemical shifts are reported relative to internal standard acetone set to 2.225 ppm (^1^H) and 30.1 ppm (^13^C).

### Methylation analysis

Methylation analysis was done by means of gas chromatography-mass spectroscopy of partially methylated alditol acetates (performed at Complex Carbohydrate Research Center at University of Georgia). Two hundred μg aliquot of the sample was permethylated following Ciucanu and Kerek [44]. The sample was treated with NaOH and methyl iodide in dry DMSO. The permethylation was repeated twice in order to completely methylate the polymer. Following the permethylation, the sample was hydrolyzed using 2 M HCl for 3 hours at 100°C. Then it was reduced with NaBD_4 _and acetylated using acetic anhydride/pyridine. The resulting partially methylated alditol acetates were analyzed on HP 5890 GC connected to mass selective detector in ESI ionization mode. The separation was done on 30 m Supelco DB-1 2330 bonded phase fused silica capillary column (0.25 mm ID).

### Transposon mutagenesis

Conjugations were performed between *V. cholerae *NRT36S as the recipient strain and *E. coli *S17λpir/putKm2 as the donor strain. Ten μl overnight culture of *V. cholerae *NRT36S was spotted on LB agar and let dry, 10 μl overnight culture of S17λpir/putKm2 was then spotted on top. After overnight incubation at room temperature, the mixture was re-suspended in 1 ml of LB broth; 50 μl of the suspension was plated onto LB agar with kanamycin and polymyxin B to select for *V. cholerae *mutants.

### DNA analysis

DNA flanking the transposon in the mutants was amplified and sequenced by a modified inverse PCR protocol [45]. Genomic DNA was isolated with PrepMan™ (Applied Biosystems) according to the manufacturer's instruction. Two μl genomic DNA of the above preparation was digested with 5 units of *Nla *III (New England Biolab) in a 20 μl reaction overnight followed by denaturing at 65°C for 15 minutes. Two μl of the digested DNA was self-ligated with 5 units of T4 DNA ligase (Invitrogen) in a 10 μl reaction. One μl of this solution was used as PCR template. Two primers were designed to anneal to the transposon mini-Km2, pointing outwards to amplify the flanking sequence of the mutant genomic DNA. The sequences of the primers are, L8 (Reverse), GTACCGAGCTCGAATTCGGCCTAG; and L9 (forward), GGAGAAAACTCACCGAGGCAGTTC. PCR was performed in a 30 μl reaction containing 100 μM of each dNTP, 1.5 mM of MgCl_2_, 1x PCR buffer (Invitrogen) and 1 unit of Taq DNA polymerase (Invitrogen). PCR products were purified with the Multiscreen PCR plates (Millipore) and sequenced using the BigDye^® ^Terminator v3.1 Cycle Sequencing Kit (Applied Biosystems). The resulting fragments were separated and recorded in an ABI 3730 × l automatic sequencer (Applied Biosystems). DNA sequence was then analyzed by the PHRED, PHRAP and CONSED software [46-48].

### Complement resistance and EM

Translucent colonies were challenged with complement in human serum as previously described [19]. EM was also performed as described [19].

### SEC

Capsule preps were analyzed by SEC using a Beckman Coulter 32 Karat HPLC, with TSK gel column (JOSOHAAS; G3000SWxL; 10 um; 30 cm × 7.5 mm), and detected at 200 nm wavelength. Purified NRT36S capsule was the same sample as used for NMR and methylation analyses. Capsule preps were prepared as followed: 10^9 ^cells were harvested into 0.5X PBS and shaken for 2 hours in a rotary shaker at 250 rpm followed by centrifuge at 12000 g for 20 min. The supernatant was treated with Dnase I and Rnase, followed by protease. The supernatant was then extracted with phenol-chloroform and precipitated with ethanol. The pellet was resuspended in water and 1/3 of the amount was loaded.

### Immuno blotting

Immuno blotting was performed as described [23]. Circa 5 × 10^6 ^bacterial cells were treated with DNase I, RNase followed by protease. Washed whole cell lysates were run on 16% SDS-polyacrylamide gel and transferred to Immun-Blot PVDF membrane (BioRad, Hercules, CA). Blots were blocked in PBS containing 3% non-fat dry milk and then incubated for 1 h in 1:1000 rabbit antiserum specific for *V. cholerae *NRT36S. The blots were washed three times with PBS and incubated with alkaline phosphatase-conjugated goat anti-rabbit immunoglobin G (Sigma) at 1:10,000 in PBS for 1 h. The blot was washed five times with PBS and developed with Western Blue colorimetric detection solution (Promega).

### Sequencing of *V. cholerae *NRT36S genome

The genome of NRT36S was sequenced by the company 454 Life Science (454 Life Science, Branford, CT) [45]. The contigs of the draft genome was compared and aligned to the fully sequenced genome of *V. cholerae *N16961 [24] by Blastn [49]. Gaps between contigs were filled only for the capsule biogenesis region, which contained the genes identified by transposon mutagenesis. Primers were designed for PCR to amplify the fragments of the gaps. PCR products were then sequenced.

### Sequence analysis and annotation

Open reading frames were predicted by the program GLIMMER[48] using the DasSarma Laboratory Autoannotation Pipeline (DLAP) (. DasSarma et al., manuscript in preparation). The settings in Glimmer were as in default, with the minimum gene size to be 90 bps and overlapping to be less than 30 bps. BlastX program [49]was used for a similarity search against the protein database in NCBI. We also used Artemis [50] to edit and confirm the results of GLIMMER.

## Abbreviations

CPS – capsule polysaccharide, LPS – liposaccharide, PCR – polymerase chain reaction, PBS – phosphate buffered saline, SDS – sodium dodeceyl sulfate, SEC size exclusion chromatography, HPLC high pressure liquid chromotagraphy, EM – electron microscopy, NMR nuclear magnetic resonance, HSQC – heteronuclear single quantum correlation, COSY – correlation spectroscopy, NOESY – nuclear Overhauser spectroscopy, TOCSY – total correlation spectroscopy, HMBC – heteronuclear multiple bond coherence

## Authors' contributions

YC performed bacteriological, genetic and EM studies, helped with the experimental design and drafted the manuscript. JA, PB, CAB performed the capsule isolation, NMR and gas chromatography studies. AA was involved in the mutagenesis studies. PP was involved in the EM and antibody studies. JAJ was involved in the microbiological experiments and experimental design. JGM was involved in the experimental design. OCS was involved in the sequencing and annotation and experimental design. All authors read and approved the final manuscript.

## Figures and Tables

**Table 1:** Results of methylation analysis.

Glycosyl residue	Percentage present
3-linked Rha	25
terminal Gal	4
3,4-linked Rha	2
3-linked Gal	13
4-linked Gal	8
4-linked Glc	3
2- and 4-linked Gal	3
3- and 4-linked Gal	4
3- and 6-linked Gal	4
4- and 6-linked Gal	1
Terminal GlcNAc	2
4-linked GlcNAc	17
4- and 6-linked GlcNAc	14

**Table 2:** Complete assignment of the NMR spectra of the de-O-acetylated polysaccharide

NRT36 (deOAc) ~50°C (321 K)	H-1	H-2	H-3	H-4	H-5	H-6, H-6'
	
	C-1	C-2	C-3	C-4	C-5	C-6
A: α-D-GlcA	5.19	3.66	3.67	3.50	3.97	-
	101.9	72.7	73.8	72.7	74.1	176.7
B: α-D-GlcNAc	5.01	3.93	4.05	3.64	4.16	4.09, 3.71
	95.5	53.9	72.0	80.2	69.9	68.6
C: β-L-Rha	4.85	4.16	3.61	3.42	3.40	1.33
	101.3	68.6	79.1	71.2	72.9	17.6
D: β-D-GlcNAc	4.42	3.78	3.67	3.67	3.49	3.83, 3.92
	102.3	56.3	74.7	77.6	75.3	61.7

**Table 3:** Residue linkages for the capsular polysaccharide of *V. cholerae *NRT36S

Linkage	HMBC	NOESY
A-B	AC1-BH4	
	AH1-BC4	
B-C	BH1-CC3	BH1-CH2
		BH1-CH3
C-D	CH1-DC4	CH1-DH4
D-B		DH1-BH6
		DH1-BH6'

**Table 4:** Complete assignment of the NMR spectra of the mono-O-acetylated polysaccharide

NRT36 Mono-O-Acetyl ~50°C	H-1	H-2	H-3	H-4	H-5	H-6, H-6'
	
	C-1	C-2	C-3	C-4	C-5	C-6
A: α-D-GlcA	5.15	3.63	3.67	3.51	3.95	-
	101.9	72.7	73.8	72.7	74.1	176.7
B: α-D-GlcNAc	4.99 (5.01)	3.91 (4.05)	3.99 (5.27)	3.63 (3.87)	4.14	4.09, 3.71
	94.9 (94.1)	53.9 (52.6)	71.6 (74.3)	80.3	68.6	68.4
C: β-L-Rha	5.04	5.53	3.80	3.48	3.49	1.36
	99.9	69.9	76.3	71.6	73.2	17.6
D: β-D-GlcNAc	4.39	3.77	3.66	3.69	3.43	3.90, 3.88
	102.1	56.3	74.7	77.8	75.1	61.7

a.) Shifts enclosed in parentheses are those of the 3-O-acetylated B residue in the native polysaccharide.						

assignment of NAc's, OAc's	NH	C = O	CH_3_			

B: α-D-GlcNAc	7.79	174.9	22.93; 2.03			
D: β-D-GlcNAc	8.09	175.2	23.19; 2.08			
C: β-L-Rha **OAc**		173.9	21.26; 2.21			

**Table 5:** CPS genes (bold print) identified by transposon mutagenesis

Clone #	Gene in CPS region	Putative gene functions	Best hit (AAI)
TR2	not	CYS regulon transcriptional activator	*V. cholerae *(100%)
TR23	not	FadR fatty acid metabolism regulator protein	*V. cholerae *(100%)
TR286	not	Adenylate cyclase	*V. cholerae *(100%)
TR301	not	Ubiquinol cytochrome C reductase	*V. cholerae *(100%)
**TR3**	**Orf23 (*galE*)**	**Nucleoside-diphosphate sugar epimerase **	***V. cholerae*** **(98%)**
**TR17**	**Orf8**	**Glycosyltransferase **	***Nitrosospira multiformis*** **(40%)**
**TR43, TR287**	**Orf43**	**Rhamnosyltransferase**	***Shewanella sp*. (66%)**
**TR296**	**Orf5 (*wzm*)**	**ABC transporter system integral membrane protein**	***Raoultella terrigena*** **(55%)**

**Table 6:** Genes in the CPS/O-antigen biogenesis region

**orf**	**symbol**	**annotated**	%AA identity/positive	species	E-Value
0	*gmhD*	ADP-L-glycero-D-mannoheptose-6-epimerase	99/99	*Vibrio cholerae*	1.00E-180
1	*rmlB*	DTDP-D-glucose-4,6-dehydratase	99/99	*Vibrio cholerae*	0
2	*rmlA*	Glucose-1-phosphate thymidylyltransferase	100/100	*Vibrio cholerae*	1.00E-167
3	*rmlD*	DTDP-6-deoxy-L-mannose-dehydrogenase	99/99	*Vibrio cholerae*	1.00E-169
4	*rmlC*	DTDP-6-deoxy-D-glucose-3,5-epimerase	100/100	*Vibrio cholerae*	1.00E-104
5	*wzm*	ABC transporter system integral membrane protein	55/77	*Raoultella terrigena*	1.00E-81
6	*wzt*	ABC transporter system ATPase component	43/59	*Nitrosospira multiformis*	8.00E-86
7		glycosyltransferase	32/49	*Rubrobacter xylanophilus*	2.00E-24
8		glycosyltransferase	40/57	*Nitrosospira multiformis*	1.00E-125
9		glycosyltransferase	41/57	*Burkholderia fungorum*	1.10E-127
10		glycosyltransferase	38/56	*Burkholderia sp*.	8.00E-56
11		dTDP-glucose-4-keto-6-deoxy-D-glucose reductase	39/64	*Actinobacillus actinomycetemcomitans*	4.00E-21
12		probable acetyl transferase by domain	30/46	*Cellulophaga sp*.	0.049
13		hypothetical protein			
14		3-hydroxybutyryl-CoA dehydrogenase	48/68	*Pseudoalteromonas haloplanktis*	2.00E-70
15		Hypothetical protein			
16		glycosyltransferase	27/44	*Syntrophus aciditrophicus*	9.00E-06
17		Hypothetical protein			
18		hypothetical protein			
19		putative glycosyl transferase	55/69	*Pseudomonas fluorescens*	8.00E-97
20		UDP-N-acetylglucosamine 2-epimerase	67/82	*Yersinia intermedia*	1.00E-147
21		glycosyltransferase	96/97	*Vibrio cholerae*	2.00E-93
22		nucleoside-diphosphate sugar epimerase	99/99	*Vibrio cholerae*	0
23	*galE*	UDP-glucose 4-epimerase VC0262	98/99	*Vibrio cholerae*	0
24	*wbeW*	galactosyl-transferase VC0263	99/99	*Vibrio cholerae*	4.00E-91
25		Trypsin-like serine proteases	96/97	*Vibrio cholerae*	1.00E-123
26	*wbfB*	hypothetical protein	99/99	*Vibrio cholerae*	0
27	*wbfC*	hypothetical protein wbfC, periplasmic	97/97	*Vibrio cholerae*	1.00E-141
28	*wbfD*	hypothetical protein wbfD	98/98	*Vibrio cholerae*	1.00E-112
29		hypothetical protein	52/58	*Vibrio cholerae*	6.00E-12
30	*wza*	Periplasmic protein involved in capsular polysaccharide export	67/82	*Vibrio splendidus*	1.00E-151
31	*wzb*	Protein-tyrosine-phosphatase	75/88	*Vibrio vulnificus*	6.00E-59
32	*wzc*	Putative tyrosine-protein kinase Wzc	75/87	*Vibrio vulnificus*	0
33	*rmlB*	dTDP-D-glucose-4,6-dehydratase	98/98	*Vibrio cholerae*	0
34	*rmlA*	glucose-1-phosphate thymidylyltransferase	100/100	*Vibrio cholerae*	1.00E-167
35	*rmlD*	dTDP-6-deoxy-L-mannose-dehydrogenase	99/99	*Vibrio cholerae*	1.00E-169
36	*rmlC*	DTDP-6-deoxy-D-glucose-3,5-epimerase	92/94	*Vibrio cholerae*	1.00E-88
37		O-acetyltransferase	38/58	*Enterococcus faecalis*	8.00E-17
38	*wzx*	O-antigen translocase	30/48	*Pelodictyon luteolum*	2.00E-39
39		glucosyltransferase	34/53	*Pseudoalteromonas tunicate*	1.00E-39
40	*wzy*	Putative saccharide polymerase			
41		Putative sugar acetyltransferase	51/72	*COG0110, Cytophaga hutchinsonii*	7.00E-43
42		glycosyltransferase	29/53	*Cytophaga hutchinsonii*	5.00E-41
43		Rhamnosyltransferase	66/77	*Shewanella sp*.	1.00E-113
44	*Ugd*	UDP-glucose 6-dehydrogenase	81/90	*Vibrio sp*.	0
45	*wecA*	Undecaprenylphosphate N-acetylglucosamine 1-phosphate transferase	96/99	*Vibrio cholerae*	1.00E-157
46		Hypothetical protein	96/96	*Vibrio cholerae*	2.00E-63
47	*rjg*	Predicted exonuclease of the beta-lactamase fold involved in RNA processing	98/99	*Vibrio cholerae*	0
